# An optimized method using peptide arrays for the identification of *in vitro* substrates of lysine methyltransferase enzymes

**DOI:** 10.1016/j.mex.2018.01.012

**Published:** 2018-01-31

**Authors:** Elyn M. Rowe, Kyle K. Biggar

**Affiliations:** Institute of Biochemistry and Department of Biology, Carleton University, 1125 Colonel By Drive, Ottawa Ontario, K1N 5B6 Canada

**Keywords:** Highly sensitive *in vitro* SPOT methylation assay, proteomics, non-histone methylation, SMYD3, MAP3K2, VEGFR1

## Abstract

While a number of post-translational modifications (PTM), such as phosphorylation and ubiquitination, have been extensively studied, lysine methylation is emerging as an important PTM with implications in a growing number of diverse cellular processes. To date, there are approximately 5000 identified methylation sites on non-histone proteins, and as the methyllysine proteome expands it becomes important to identify the lysine methyltransferase enzymes responsible for each methylation event. The use of peptide SPOT methylation assay has proven to be a useful in the identification and validation of novel substrates for lysine methyltransferase enzymes as it uses a weak beta emitter coupled with fluorography to detect methylation events. The method described in this paper provides improvements to the typical protocol for this assay, as a highly sensitive tritium assay can be developed with less radioactivity than previously described. This protocol provides an inexpensive alternative to weak beta signal enhancer sprays and washes for use in lysine methylation peptide SPOT arrays, and a simple open-source method for array quantification.

## Graphical abstract

**Figure:**
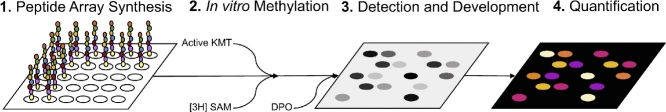

**Specifications Table**

**Table:** 

Subject area	Biochemistry, Genetics and Molecular Biology
More specific subject area	proteomics, lysine methylation
Protocol name	Highly sensitive *in vitro* SPOT methylation assay
Name and reference of original method	*Adapted from:* S. Kudithipudi, D. Kusevic, S. Weirich, A. Jeltsch. Specificity analysis of protein lysine methyltransferases using SPOT peptide arrays. J Vis Exp., 93 (2014)
Resource availability	• Adenosyl-l-methionine, S-[methyl-3H] (Perkin Elmer, Cat# NET155V250UC)
	• 2,5-Diphenyloxazole (DPO) (Sigma-Aldrich, Cat# D210404)
	• Amersham Hyperfilm MP (GE Healthcare Life Sciences, Cat# 28906842)
	• ImageJ software (available at http://imagej.nih.gov/ij/)

## Value of the Protocol

**Table:** 

• Simple method to identify *in vitro* peptide substrates and determine substrate specificity of lysine methyltransferase (KMT) enzymes
• Optimized concentration of radioactivity for a safer and more cost-efficient method
• Effective enhancer alternative described for expensive tritium enhancer sprays for array-based fluorometry assays
• Open-source array quantification for experimental analysis

## Protocol background

It is well-established that lysine methyltransferase (KMT) enzymes play a role in the methylation of histones and in effect, the regulation of gene expression in the nucleus. In recent years, however, it has become evident that these enzymes play a more complex role in the cell, as approximately 5000 methylation sites on non-histone proteins have been identified. Since only approximately 50 KMTs have been identified, it would appear that each one is capable of methylating a fairly large subset of the outlined methyllysine proteome – the question lies in which enzymes are methylating which substrates. Furthermore, it has been demonstrated that some KMTs (e.g. SMYD3, EZH2, G9a, etc.) are over-expressed or dysregulated in many different types of cancer [1]. While their roles in terms of histone lysine methylation have been elucidated [2] recent research has focused on identifying novel cytoplasmic substrates and investigating possible non-histone mechanisms of action that propagate the diseases. The SPOT methylation array using adenosyl-l-methionine, S-[methyl-3H], first used in 2008 by Rathert et al. [3], has proven to be an effective method for identifying new substrates of KMTs and is now widely used. While effective weak beta emitter enhancer sprays and washes are commercially available, the method described below provides an inexpensive alternative for producing clear images of radioactively labelled peptide arrays using 2,5-diphenyloxazole (DPO). This method also provides the optimized amounts of radioactivity needed to reduce the potentially high costs of in vitro methylation assays, and is able to use a lower concentration of radioactivity than previously reported [4].

## Method details

### Step 1: preparation of peptide SPOT arrays

Custom peptide arrays can be synthesized using Fmoc chemistry with specialized equipment (e.g., Intavis ResPep SL) directly to aminated cellulose membranes [5]. As a low cost alternative to purchasing commercially available membranes, the aminated cellulose membrane can also be prepared in lab prior to peptide synthesis [6]. Following synthesis and deprotection of peptides, the arrays can be washed 6× with dichloromethane (DCM), followed by two washes with anhydrous ethanol. Membranes are then dried overnight in a desiccator and can then be used for methyltransferase assays.

### Alternative step 1: preparation of peptide dot blots

#### Materials

• Blocking buffer (50 mM Tris [pH 7.4],150 mM NaCl, 0.05% tween 20, 5% nonfat skim milk)
• TBS-T buffer (50 mM Tris [pH 7.4], 150 mM NaCl, 0.05% tween 20)
• Nitrocellulose membrane (Sigma Aldrich, Cat# N8520)
• 5 mM Peptide

#### Procedure

1. Spot 1 μL of substrate peptide onto a nitrocellulose membrane for a total of 5 nmol of peptide per spot.
2. Allow peptides to air dry on the nitrocellulose membrane.
3. Block array for 1 h with blocking buffer on a rocking platform at RT.
4. Wash array once with TBS-T buffer for 5 min on a rocking platform.
5. Array can be stored (or frozen at −20C) in TBS-T for future use.

### Step 2: in vitro methylation

#### Materials

• Methylation buffer (50 mM Tris [pH 8.5], 2 mM MgCl_2_, 1 mM dithiothreitol (DTT))

**Note**: *DTT should be added immediately before use as it oxidizes in solution*

• Adenosyl-L-methionine, S-[methyl-3H] (i.e., [3H]SAM) (Perkin Elmer, Cat# NET155V250UC)
• Active KMT enzyme
• Plastic container with lid

#### Procedure

(1) Peptide array is initially incubated in 6 mL of methylation buffer, without added [3H]SAM or KMT enzyme, for 10 min
(2) Remove the 6 mL of methylation buffer from the array and add 6 mL of fresh methylation buffer with 0.466 μM [3H]SAM (specific activity: 80Ci/mmol) and 0.1 μM–1 μM of active KMT enzyme to the array. The amount of active KMT added will depend on the activity of the KMT. Typically, 0.5 μM is a good place to begin assays with.

**Note**: *The outlined procedure uses volumes and equipment appropriate for an 8.5* *cm × 6* *cm peptide array. Volumes and incubation box should be adjusted based on array size, such that the array is fully submerged in liquid during incubation.*

(3) Lid the container and seal with parafilm prevent evaporation during incubation.
(4) Leave the array to incubate overnight on a rocking platform at room temperature.

### Step 3: detection and development

While there are commercially available means of enhancing and detecting the weak radioactivity emitted by tritium such as enhancing sprays and phosphor screens, they are rather pricey. In the place of these products, a solution of 7% 2,5-Diphenyloxazole (DPO) in ethanol, in combination with intensifying screens, can be used and have been shown to effectively enhance the radioactive signal and produce clear film images of peptide arrays. If available, phosphor screens and a phosphoimager can be used in the place of the film described, as sensitivity can be higher and the exposure time can potentially be reduced.

#### Materials

• Wash Buffer (100 mM NH_4_HCO_3_, 1% sodium dodecyl sulfate (SDS))
• 2,5-Diphenyloxazole (DPO) (Sigma-Aldrich, Cat# D210404)
• Ethanol, anhydrous
• Forceps
• Plastic food wrap
• Film developer solution (e.g., Ilford ilfosol 3, Cat# 1131778)
• Film fix solution (e.g., Ilford rapid fixer, Cat# 1984253)
• Amersham Hyperfilm MP (GE Healthcare Life Sciences, Cat# 28906842)
• Radiographic film cassette
• Carestream BioMax TransScreen LE Intensifying screens (Fisher Scientific, Cat# 05-728-67)

#### Procedure

**Fig. 1:**
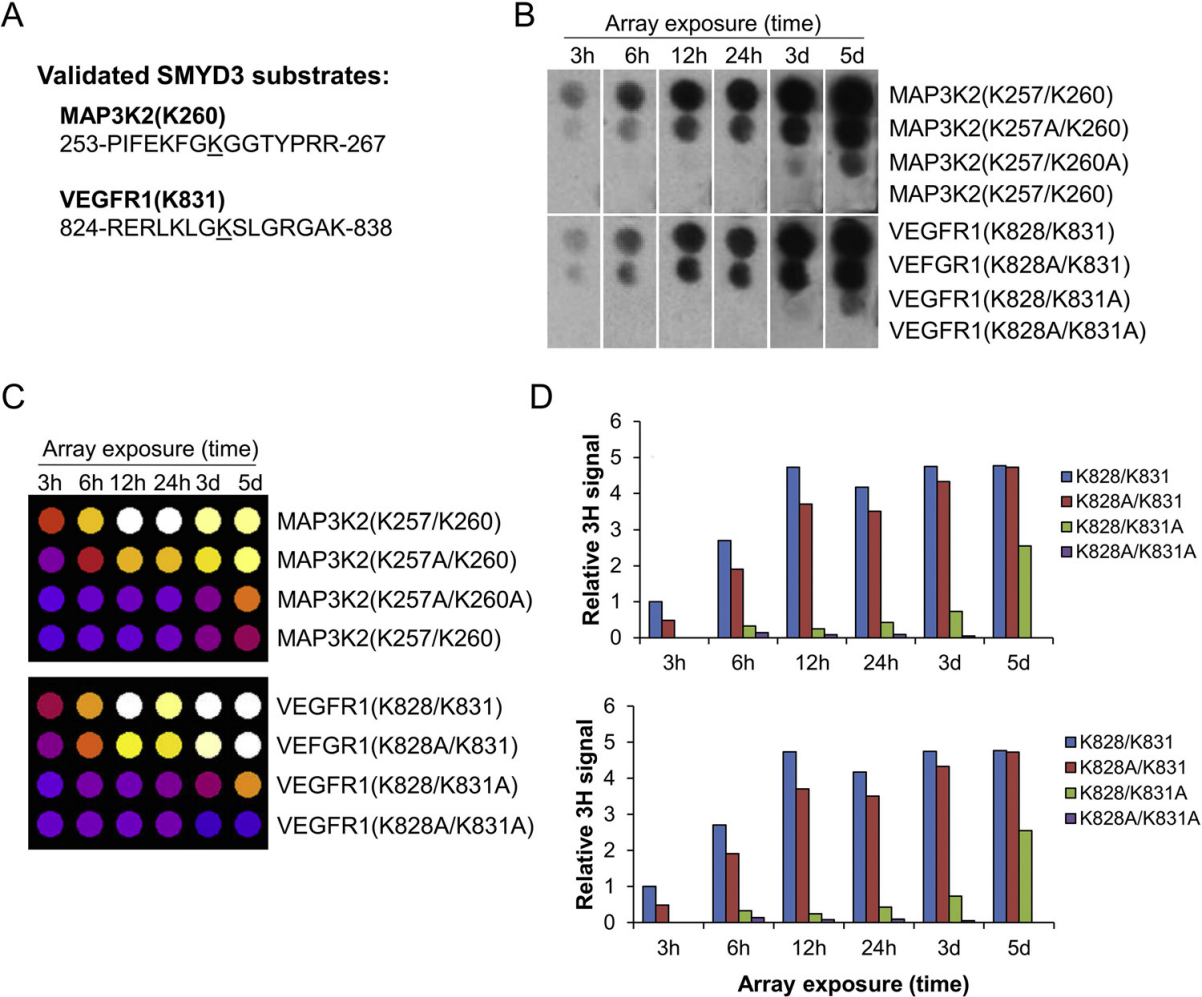
SMYD3 methylation determined by in vitro substrate arrays. A) The amino acid sequences of two validated SMYD3 substrates. The lysine-specific methylation site is underlined and central to the substrate peptides. B) Methylation array exposure times on for both the MAP3K2 and VEGFR1 validated SYMD3 substrate peptides. C) Visual representation image of digitized SPOT array used for quantification of SPOT intensity. Analysis of SPOT intensity, and SMYD3 substrate activity, was determined by densitometry of the 3H signal. D) Histograms show relative quantification of 3H signal as detected from the SPOT arrays at different exposure times.

1. Prepare a 7% DPO solution by dissolving 7 g of DPO in 100 mL ethanol (this mixture can be stored at room temperature for later use). This will solution will be used later in the procedure to enhance the 3H signal from the array.
2. Next, remove the methylation buffer from array into a labeled radioactive waste storage container for later disposal.
3. Add 10 mL of wash buffer to the array and incubate the array on a rocker for 3 min at RT.
4. Repeat wash step with fresh wash buffer 5× for 3 min
5. Following washing of the array, using forceps transfer the array to a new containing enough anhydrous ethanol to completely submerge the array. Incubated in ethanol on a rocking platform for 2 min.
6. Following incubation of the array in ethanol, remove the ethanol and enough of the 7% DPO solution to complete submerge the array. Gently agitate for 2 min to ensure uniform distribution of the DPO solution.
7. Remove the array from the DPO solution and allowed to dry completely on filter paper (approximately 3 h or overnight) prior to array fluorography.
8. Prepare the radiographic film cassette by attaching intensifying screens (Carestream BioMax TransScreen LE) to both the inner bottom and inner cover of the cassette. This will help to enhance the 3H signal from the array.
9. Carefully wrap the dry array with a single layer of plastic wrap. Place the array into the cassette.
10. In complete darkness, remove a sheet of detection film (Amersham Hyperfilm MP) and cut one corner to maintain proper orientation in future analysis. Add the film to the cassette along with the prepared array.
11. Close the cassette and expose the film to array fluorography at −80 °C. Exposure time will be dependant on the activity of the KMT enzyme and substrate concentration. Most array exposures will be in the range of hours to days at −80 °C. It is recommended to carry out an initial 24 h exposure and adjust exposure time from the obtained results.
12. Developer and fixer were prepared according to package instructions and the film was developed under a safe light (Fig. 1b).

### Step 4: quantification

In most cases, specialized software is required for the detection and quantification of array-formatted images. To observe and quantify the peptide array, a free open-source program can be used for efficient and reliable spot quantification to compare intensity values.

#### Materials

• Film imager (e.g., Bio-Rad GS-800 calibrated densitometer)
• Computer with the ImageJ program installed (http://imagej.nih.gov/ij/)

#### Procedure

1. Open the ImageJ program and install the ‘Protein Array Analyzer’ macro by clicking on the “toolsets” menu icon of the ImageJ tool bar. If this is not available, download the macro directly (https://imagej.nih.gov/ij/macros/toolsets/) and following instructions to install.
2. Open the array image within the ImageJ program. Click the “Array Analysis Menu” and select “Array Analysis” menu to display the graphical interface required to analyze the opened array image. This action also proposes a method of background correction (none, liner, or paraboloid). It is suggested to use default settings for background subtraction.
3. Within the interface, optimize the visualization by activating some options available such as setting the diameter of the array spots for analysis. The spot diameter must be properly set, such that the automatically positioned spots of the array do not overlap. Click anywhere within the array and adjust the ‘+’ and ‘−’ buttons in the left-most icon to change the spot size. The circle radius defines the quantification area.
4. Set the number of columns (nCol.) and rows (nLin.) you wish to analyze in the array image.
5. Located to the right of the nLin icon, set the three required locations (top-left, top-right, and bottom left) using the respective icons. To set a mark, first click on one of the (first) top-left, (second) top-right, or (third) bottom-left icons, then position on a cross hair icon initially located in the top-left of the screen and drag the cursor to the correct location corresponding to your selection (i.e., top-left, top-right, or bottom left). Complete this for all three positions individually to coordinate the three corners of the array. Icons will turn from ‘red’ to ‘green’ once set.
6. The interface will then automatically place outlines around your spots to be analyzed. If adjustments are required, simply readjust the marker positions.
7. Once all spots are appropriately positioned, click the “Model” button to obtain the array quantification to be used for analysis and a modeled image of the array (Fig. 1C).

### Method validation

The described method was used to effectively two known and well-studied non-histone substrates of SMYD3; a KMT that has been found to be over-expressed in many types of cancer [7]. While some of this KMT’s action in the propagation of cancer has been elucidated, including its methylation of MAP3K2 contributing to Ras-driven cancer [8], much remains unknown about its action in the cell. Fig. 1 displays the data obtained from this method for the two previously identified non-histone substrates – MAP3K2 and VEGFR1. Since both of the substrate peptides contain multiple lysine residues that may also be subject to SMYD3-induced methylation in vitro (i.e., passenger lysine) (Fig. 1A), four variations of the peptides were synthesized on each array to act as methyl-site specific controls – with both lysine residues present, with either the passenger lysine or central lysine mutated to an alanine residue, and with both lysine residues mutated to alanine (Fig. 1B). As shown in Fig. 1D, an exposure time of 3–5 days resulted in a weak signal from the passenger lysine, demonstrating the importance of proper optimization of array exposure. An exposure time of 24 h was found to be optimal for this particular array. It should be noted that this method can be adapted for a wide variety of methyltransferase enzymes, and other arrays utilizing tritium detection methods.
